# Automated colonoscopy withdrawal phase duration estimation using cecum detection and surgical tasks classification

**DOI:** 10.1364/BOE.485069

**Published:** 2023-05-12

**Authors:** Thomas De Carvalho, Rawen Kader, Patrick Brandao, Juana González-Bueno Puyal, Laurence B. Lovat, Peter Mountney, Danail Stoyanov

**Affiliations:** 1 Odin Vision, London, UK; 2 Wellcome/EPSRC Centre for Interventional & Surgical Sciences, University College London, London, UK; 3 Division of Surgery & Interventional Science, University College London, London, UK; 4 Gastrointestinal Services, University College London Hospital, London, UK

## Abstract

Colorectal cancer is the third most common type of cancer with almost two million new cases worldwide. They develop from neoplastic polyps, most commonly adenomas, which can be removed during colonoscopy to prevent colorectal cancer from occurring. Unfortunately, up to a quarter of polyps are missed during colonoscopies. Studies have shown that polyp detection during a procedure correlates with the time spent searching for polyps, called the withdrawal time. The different phases of the procedure (cleaning, therapeutic, and exploration phases) make it difficult to precisely measure the withdrawal time, which should only include the exploration phase. Separating this from the other phases requires manual time measurement during the procedure which is rarely performed. In this study, we propose a method to automatically detect the cecum, which is the start of the withdrawal phase, and to classify the different phases of the colonoscopy, which allows precise estimation of the final withdrawal time. This is achieved using a Resnet for both detection and classification trained with two public datasets and a private dataset composed of 96 full procedures. Out of 19 testing procedures, 18 have their withdrawal time correctly estimated, with a mean error of 5.52 seconds per minute per procedure.

## 1. Introduction

**Fig. 1.**
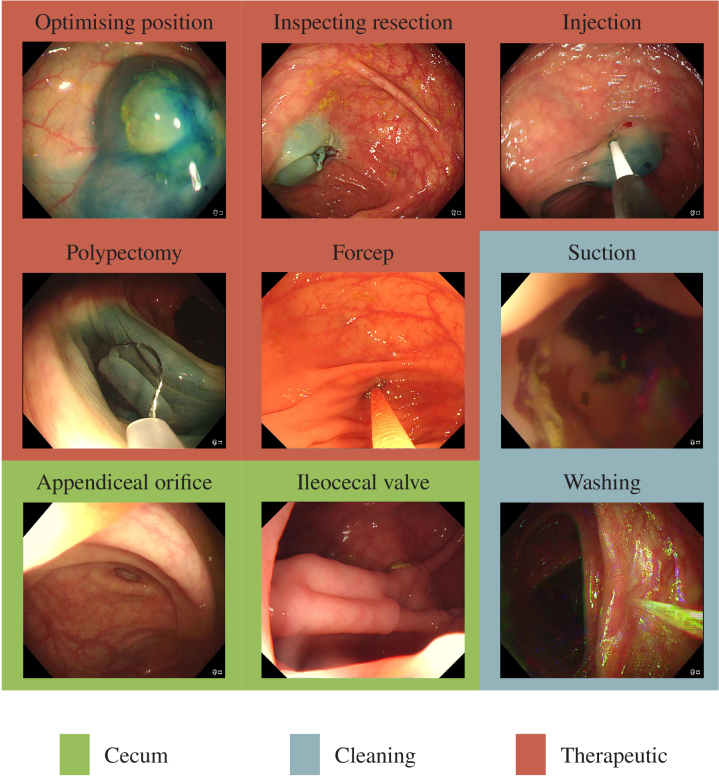
Examples of frames from each colonoscopy phase class and subclass. The data is labelled for each of the subclasses, but some of them can be regrouped under the same class label (for example washing and suction are both cleaning phases). The grouped class labels are used to train and test the network.

**Fig. 2.**
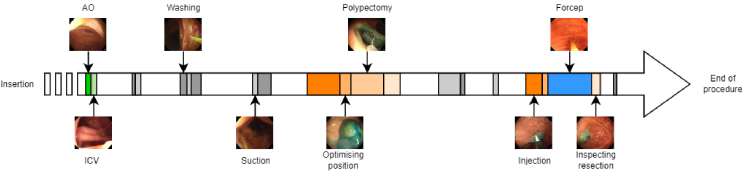
Example of the timeline of a procedure. The cleaning phases can happen at any time and are usually quite short. The AO and the ICV mark the beginning of the withdrawal phase and are usually found together. The therapeutic phases are following each other in this order most of the time but this can always vary depending on the procedure.

With almost two million new cases around the world, colorectal cancer is the third most common type of cancer according to the world cancer research fund international. During a colonoscopy procedure, the gold standard for diagnosing colorectal cancer, the bowel is inspected to find pre-cancerous lesions called polyps. Each missed polyp, especially adenomas, increases the chances of developing cancer and therefore lowers the survival chances of the patient. The risk of cancer was found to decrease by 3.0% with each 1.0% increase in the adenoma detection rate [1]. Standard screening protocol requires the insertion of the scope until the cecum is reached. From this point, the scope is slowly withdrawn allowing the full inspection of the colon. Several studies establish a direct link between the withdrawal time and the polyp detection rate, [2–5]. The suggested optimal time can vary according to the study but the consensus is that the longer withdrawal time correlates to higher polyp detection. Therefore, measuring the withdrawal time is critical to quantify the quality of the procedure. The British Society of Gastroenterology recommends a minimal withdrawal time of 6 minutes and a target time of 10 minutes [6]. Real-time computation of this metric would then encourage clinicians to spend the right amount of time exploring the colon to improve the quality of colonoscopies. The biggest challenge in measuring withdrawal time is that, during a colonoscopy, other tasks are performed apart from the exploratory phase. For instance, if there is poor bowel preparation, the bowel must be cleaned during the procedure. Washing is performed with a water jet to remove the pieces of stool and suction is used to remove colonic content. These measures improve the visualisation of the colonic mucosa. Figure 1 shows the various tasks involved in colonoscopy. The typical timeline of a procedure is shown in Fig. 2. Currently, withdrawal time is measured from the detection of the cecum to the withdrawal of the endoscope from the anal canal. This measurement includes all phases of withdrawal, including exploratory, cleaning, and therapeutic phases. However, the therapeutic and cleaning phases should be ignored to measure the exact withdrawal time. Automating the detection of the phases of colonoscopy would allow accurate measurement of the real withdrawal time in any type of procedure, including those with extensive cleaning and numerous polyp resections. This would allow real-time automated feedback to the endoscopist of the withdrawal time during the procedure, for example with a timer that increments only when exploration is happening and pauses during cleaning and therapeutic phases, so that their speed of withdrawal could be adjusted accordingly. This also allows a more accurate measurement to audit endoscopists to identify those with an inadequate withdrawal time who would benefit from further endoscopic training and would therefore indirectly improve their polyp detection rate.

The direct detection of polyps during colonoscopies is an achievable feat, as evidenced by a significant number of studies conducted on this topic. Particularly noteworthy are recent investigations that have placed high emphasis on the application of deep learning techniques, as demonstrated in the works of Urban et al. [7], Wang et al. [8], Puyal et al. [9,10], Brandao et al. [11,12], and many others [13]. These studies have explored the use of detection systems as a direct means to improve the polyp detection rate. In contrast, measuring the withdrawal time during the procedure is more focused on the reporting aspect of the process and is a different but complementary approach. When implemented in real-time, through a timer for example, this approach can encourage clinicians to allocate sufficient time towards polyp detection, which in turn, may indirectly enhance their adenoma detection rate.

The withdrawal time estimation can be separated into two tasks, the cecum detection to identify the start of the withdrawal phase, and a classification task to identify the therapeutic, and cleaning phases. The final time estimation is then the difference between the total time of the procedure since the cecum is reached and the time spent in the different non-exploratory phases. Previous work has been performed for cecum detection. The appendiceal orifice (AO) and the ileocecal valve (ICV) are both parts of the cecum and can be detected independently. Cao et al. [14] propose the first detection method for the AO using hand-crafted features specific to their dataset. Wang et al. [15] improve this method using more advanced features. Wang et al. [16] later use a similar method to identify images or videos containing an appendiceal orifice. Lebedev et al. [17] improve the detection using a deep neural network based on a ResNet50. Finally, Katzir et al. [18] propose a method to detect the cecum using the AO, the ICV and a third structure called the triradiate fold. The method is a combination of landmark detection, depth estimation and egomotion. The detection of the ileocecal valve has also been explored by Summers et al. [19] but only on computerized tomography (CT) scans. Building on previous cecum detection systems, this study presents a deep-learning platform to detect the AO and the ICV simultaneously.

Previous classification task studies in colonoscopy procedures mainly concern polyp classification. However, a few studies propose an automatic method to obtain quality metrics from a colonoscopy. Hwang et al. [20] focus on 5 metrics: the insertion time, the withdrawal time, the clear withdrawal time, the number of camera motion changes, and the fraction of the clear withdrawal that is spent for close inspections of the colon wall ("wall views") and the ratio of close inspections to global inspections ("lumen views"). The starting point of the withdrawal phase, and therefore the insertion time and the withdrawal time, are computed based on the motion estimation of the camera. The clear withdrawal time consists of the withdrawal time without the out-of-focus frames which are filtered using a discrete Fourier transform, a texture analysis, and finally, a frame clustering [21]. The main difference here is that only the out-of-focus frames are ignored, while therapeutic frames for example, which usually have a very high quality, are included. Therefore, this method cannot be used to remove all non-exploratory phases from the withdrawal time estimation. Chang et al. [22] propose a deep learning method to extract quality metrics from colonoscopy procedures by classifying biopsies, tools, bowel preparation, and other phases such as withdrawal. The withdrawal phase is described there as the time interval between the first cecal image and the last colon image during each procedure. The cleaning and therapeutic phases are still included in their measurements. Moreover, the cleaning phase is not classified at all by Chang et al. [22]. Katzir et al. [18] use their cecum detection method to detect the start of the withdrawal phase, but no other phase classification is performed, making it similar to previous studies. Phase classification in colonoscopy is not a trivial problem due to the similarity between different classes. This is especially the case for the cleaning class where frames can be visually very similar to normal inspection views. The quick alternation between cleaning, withdrawal, and even therapeutic phases makes labelling and classification harder. This study has the same objective as Chang et al. [22] and Katzir et al. [18], but with a more accurate estimation of the withdrawal time, achieved by classifying and excluding the therapeutic and cleaning phases.

Similar work has been done on other surgical tasks, especially laparoscopy or eye surgery. The first proposed methods were based on hand-crafted features, [23,24], which can be very efficient but hard to generalize to a wider dataset. More advanced methods using deep learning were also explored. Twinanda et al. [25] propose an architecture to recognize the different tasks on laparoscopic videos by separating the phase recognition and the tool presence detection. Both tasks are achieved using a convolutional neural network (CNN) modified and finned-tuned for this purpose combined with a hidden Markov model (HMM). Jin et al. [26] use a more advanced architecture, keeping the same base idea of neural networks but including more temporal information using a recurrent neural network (RNN). Padoy et al. [27] shows two examples of workflow recognition during surgery using the same methods, a CNN combined with either an RNN or an HMM. Kitaguchi et al. [28] also separate tool recognition and phase classification during laparoscopy and use CNNs as well but without any HMM or RNN this time. All of these methods show that neural networks are one of the best solutions when it comes to task recognition. Currently, none of these was tested on colonoscopy procedures, where the type of phases to classify is different from pure surgical phases. For example, the cleaning phases do not involve tools, which makes them harder to recognize.

This study presents a fully automatic method to measure the withdrawal time during colonoscopies. It includes cecum detection and classification of the cleaning and therapeutic phases, which allows a precise measure of the final withdrawal time. Both of these tasks are achieved using CNNs.

For the detection task, a sensitivity of over 75% for both AO and ICV is achieved on two internal datasets and one public dataset, with a specificity of over 93% for the ICV and over 89% for the AO. Withdrawal time estimation achieves a sensitivity over 82% and a specificity over 92% for all the classes. 94.7% of the procedures are successfully classified as longer or shorter than 6 minutes with a mean error of 5.52 seconds per minute per procedure.

## 2. Methods

### 2.1 Withdrawal time estimation

This study is separated into two main tasks: cecum detection and classification of the different phases of the procedure. The cecum can be detected either by detecting the ileocecal valve (ICV) or the appendiceal orifice (AO). Both are structures that indicate that the cecum has been reached (see Fig. 1). The detection of the cecum triggers the start of the classification task. The different phases to classify are shown in Fig. 1, namely the cleaning phase and the therapeutic phase. Both of these classes are excluded from the final withdrawal time. The objective is to know if each frame belongs to one of these classes or if it is a negative one where inspection is being performed. The final withdrawal time is then defined as

(1) $$t_{final}=\frac{n_{\mathrm{total}}-n_{\mathrm{insertion}}-n_{\mathrm{cleaning}}-n_{\mathrm{therapeutic}}}{f}$$

with $t_{final}$ the final withdrawal time, $n_{total}$ the total number of frames, $n_{insertion}$ the number of frames between the beginning of the procedure and the first AO or ICV frame, $n_{cleaning}$ the number of cleaning frames, $n_{therapeutic}$ the number of therapeutic frames, and f the frame rate.

### 2.2 Cecum detection

**Fig. 3.**
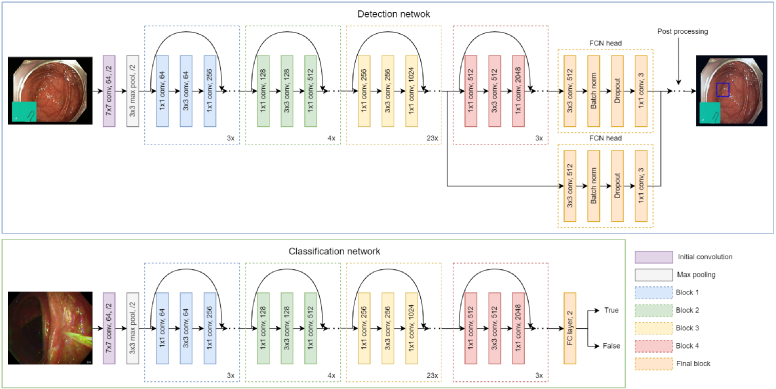
Architecture of the networks used for the detection task and the classification task. The post-processing phase transforms the raw predictions into a detection box for each channel as shown in Fig. 4. The classification network is the same for each different phase.

Cecum detection is formulated as an instance segmentation task with a multiclass approach, where each pixel of an input frame is classified as belonging to the AO, the ICV, or the background class. A fully convolutional network (FCN) [29] was implemented using a Resnet 101 backbone [30], as shown in Fig. 3. The model is pre-trained on ImageNET [31]. A cross-entropy loss is used for both the auxiliary and final layers, the auxiliary layer being a segmentation head receiving features from the third backbone building block instead of the final one. The FCN head is composed of a 3x3 convolutional layer, a batch normalization layer, a dropout layer, and a final 1x1 convolutional layer with a softmax activation function and is the same for both the final and the auxiliary layers. The total loss L is defined as: $L=L_{aux}+L_{final}$ the sum of the auxiliary loss $L_{aux}$ and the final loss $L_{final}$ (as implemented in [9] and [10]). The feature maps used to compute the auxiliary loss have undergone fewer pooling steps, which leads to larger feature maps and a higher resolution in the output compared to the final layer. The combination of the two losses allows a more precise prediction. Adagrad is used as the optimizer, with a learning rate of 0.001, and a batch size of 32 and 500 mini-epochs. The model with the best accuracy on the validation set is kept as the final model.

**Fig. 4.**
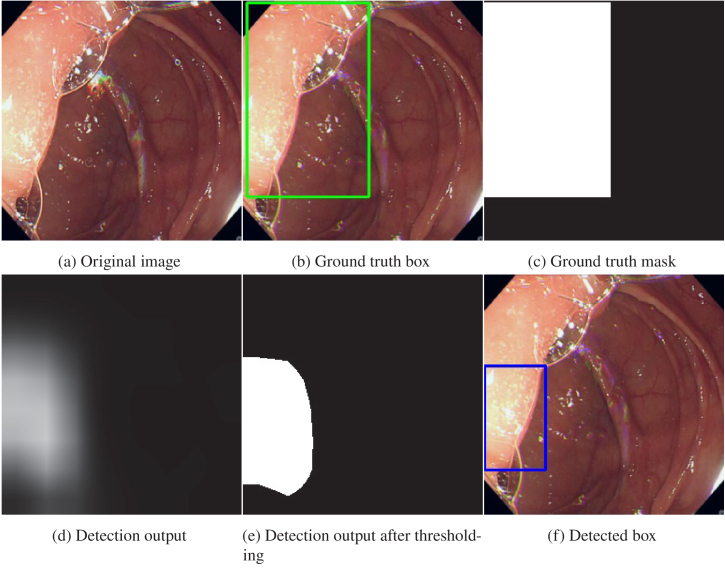
Example of ground truth and the corresponding output from the detection model for the ICV channel. The annotated box is used to create the mask and the output from the model is post-processed to obtain a detection box which is compared to the ground truth. Similar images are obtained for the AO and background channels.

**Fig. 5.**
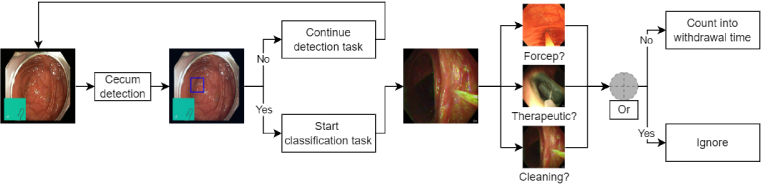
Pipeline of the detection and the classification tasks. This pipeline is applied to each frame independently. Both tasks are independent as well. The different models for the classification task are also independent but their binary prediction is combined using a logical OR.

The frames are extracted from the colonoscopy videos and used as input for the network. The frames are resized to 256 by 256 pixels and normalized using the mean and standard deviation values from ImageNET before being fed to the network. Random transformations are then applied as data augmentations, including rotation, translation, scaling, and colour transformations. The data was split into training, testing, and validating, with 70% of the procedures for training, 20% for testing, and 10% for validation, with no patient overlap. Negative frames are also used in training, testing, and validation, with a ratio of 1/3 negative and 2/3 positive. The labels are separated masks for AO and ICV to handle the case where both are present on the same frame. The model outputs a 248 by 248 by 3 pixels image, with a prediction channel per class. An example is shown in Fig. 4(d). At test time, video frames from the full insertion are evaluated consecutively. Once the cecum is detected in a frame, the cecum detection task finishes, and the phase classification tasks begin, as depicted in Fig. 5.

### 2.3 Classification of therapeutic and cleaning phases

For the classification task, a Resnet 101 pre-trained on ImageNET is used as shown in Fig. 3. The last layer is a fully convolutional layer with two classes as output, the positive and the negative one, and a softmax activation function. The positive class is composed of the non-exploratory phases, cleaning and therapeutic, and the negative class is composed of all the other frames. One binary classifier is trained for each group of labels. The model is trained with a cross-entropy loss and a learning rate of 0.01, Adagrad as an optimizer, and a batch size of 32. The model with the best accuracy on the validation set is kept. As for the cecum detection task, the frames are extracted from the colonoscopy video. They are then resized to 224 by 224 pixels and normalized using the pixel distribution values from ImageNET. The same random transformations as for the detection task are used.

The main challenge for the classification task is to handle several unbalanced classes simultaneously, with some of them very similar to each other. The split used is 70% of the procedures for training, 20% for testing, and 10% for validation. The split is created by balancing the three sets as much as possible, ensuring that the ratios between training, testing, and validation are the same for all the labels. This means that, for example, 70% of the cleaning frames will be in the training set, 20% will be in the testing set and 10% will be in the validation set. This is achieved using a k-means clustering method [32] to regroup similar procedures and then using stratified sampling to ensure that each cluster is proportionally represented in each set. The cluster splitter uses the number of frames in each of the following classes to create the clusters: AO, ICV, washing, suction, injection, position optimisation, polypectomy, resection inspection, forcep, narrow band images (NBI), and narrow-band images with near focus (NBI-NF).

Each classification network is designed to distinguish one class against all other images. For each class, all of its subclasses (for example, washing and suction for cleaning) are used as positive frames. Then the training and the validation set are balanced by subsampling each class so that the number of frames from each subclass is the same. For example, the same number of washing and suction frames is used in the cleaning class. The negative frames are the frames with any other label. For the cleaning model, it would be therapeutic and all the frames without a label. Negative frames are also added to the training and the validation set with a ratio of 2/3 negative and 1/3 positive. More negative frames are used for training and validation to increase the diversity of the negative class. For the testing set, the full video is used without any kind of subsampling. On top of the cleaning and therapeutic classifiers, a forcep classifier is also trained to improve the overall performance of the final model on the therapeutic class. The reason for this is explained in the results section.

As shown in Fig. 5, each model is used simultaneously and a logical OR is applied to the results because multiple phases can happen at the same time such as cleaning and therapeutic. If the frame is predicted as part of any of the three class groups, it will be discarded from the final withdrawal time estimation. With $P_{cleaning}$ , $P_{therapeutic}$ and $P_{forcep}$ the predictions for each model, being either true if the frame is part of the class or false otherwise, the final prediction $P_{final}$ is defined as

(2) $$P_{final}\equiv P_{cleaning}\vee P_{therapeutic}\vee P_{forcep}$$

Temporal filtering and thresholding are applied as post-processing to increase the robustness of the results. Using a sliding window of size n , a voting scheme is applied, so the final prediction for a given frame is the most represented predicted class from the frames in the window of size n centered on that frame. This smooths the results and helps remove short noisy predictions. Thresholding is also used after the temporal filter and removes the low-confidence predictions using the raw value given by the network. The threshold allows an increase in the specificity of the network.

Classifying the different phases of a colonoscopy to estimate a precise withdrawal time has not been done before and this study gives a baseline for this task. The novelty of this study is a fully automated system capable of accurately estimating the real withdrawal time. The split takes into account a lot of parameters to ensure a good distribution of the procedures. The real withdrawal time is known to be a good indicator of the quality of a procedure, but it was impossible to measure it precisely as long as some cleaning or polyp removal was happening. This study proposes a full pipeline allowing that.

## 3. Results and discussion

### 3.1 Datasets

Three datasets are used in this study. One is used for both cecum detection and phase classification and two are used only for cecum detection. An overview of the datasets is shown in Table 1.

**Table 1.** Overview of the datasets used in this study.

Dataset	Status	Number of videos	Number of frames	Number of labels
Full procedures	Private	96	3,963,030	10
Kvasir	Public	N/A	8000	2
Static frames	Private	N/A	73,876	3

**Fig. 6.**
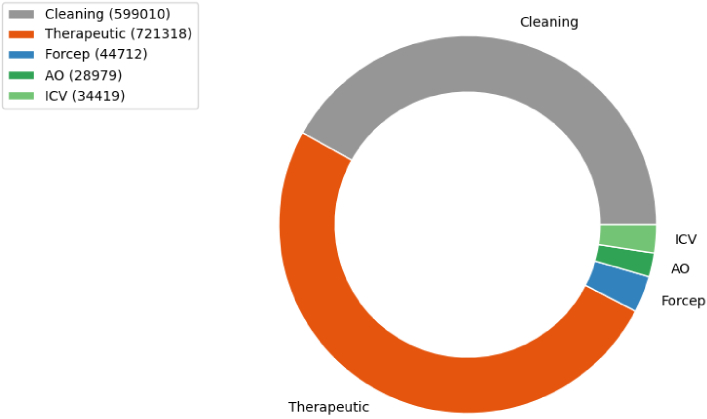
Diagram showing the distribution of the full procedures dataset. Each class used is represented along with its number of annotated frames and its group.

The dataset used for classification is composed of 96 full colonoscopy procedures acquired with an Olympus 290 scope at the University College London Hospital from April 2019 up to August 2021. The study was approved by the Cambridge central research medical ethics committee (REC Reference No.18/EE/0148). Each procedure is performed on a different patient and is fully labelled by an expert. The total number of frames is 3,963,030. The procedure videos were labelled for 10 different labels. The classes are very unbalanced, as shown in Fig. 6, with a small number of forcep, AO and ICV frames compared to cleaning and therapeutic ones. Some examples of the labels are shown in Fig. 1. They are also sufficient to obtain a precise withdrawal time estimation for all of the procedures. The frames were labelled one by one with the corresponding classes by a gastroenterology doctor with expertise in artificial intelligence applied to colonoscopy. The labels are divided into 2 groups, one for each of the main phases of a colonoscopy. The first group, cleaning, is composed of suctioning of colonic content ("suction") and washing of the colonic mucosa ("washing"). The second group is the therapeutic group and contains the labels ’injection’ (defined as the injection of fluid content to lift the polyp from the submucosa), optimising position (defined as the phase between finishing injection and starting polypectomy), "polypectomy" (defined as removal of the polyp using a surgical instrument), inspecting resection (defined as inspecting the resection margins of the polyp for any residual polyp) and "forcep" (commonly used for routine biopsies). A frame can have two labels at the same time but, since a different model is trained for each group, this just means that the frame will be used as a positive frame for both models.

For the AO and the ICV, bounding boxes around the corresponding structures are used as shown in Fig. 4(b). The clinician labels each frame with a bounding box and the coordinates are then used to create a binary mask which is used to train the segmentation model, as depicted in Fig. 4(c). The frames containing an AO or an ICV were labelled either low or high quality depending on the quality of the view and the presence of artefacts on the image. For the AO and ICV detection, two other datasets are also used for training. The Kvasir dataset [33] is a public dataset containing various labels including some cecum. There are 1000 cecum images and 7000 images with other various labels. Only the cecum class can be used as positive frames for the segmentation model but the others are used as negative images. The cecum class is composed exclusively of AO images with no ICV examples. The second dataset is a set of 73,876 static frames from the University College London Hospital (UCLH). It contains both AO and ICV and some other classes like polyps which are used as negative images. There are 1,158 frames containing ICV and 778 frames containing AO. Both of these datasets are only used for the AO/ICV detection task.

### 3.2 Evaluation metrics

The metrics used for the evaluation of the models are sensitivity, specificity, and time to detection. The sensitivity or true positive rate TPR is defined as

(3) $$\mathrm{TPR}=\frac{TP}{TP+FN}$$

with TP the number of true positives and FN the number of false negatives and measure how good the network is at classifying positive frames. The specificity or true negative rate TNR is defined as

(4) $$TNR=\frac{TN}{TN+FP}$$

with TN the true negatives and FP the false positives and measure how good the network is at classifying negative frames. The time to detection $t_{detection}$ is defined as

(5) $$t_{detection}=t_{model}-t_{groundtruth}$$

with $t_{model}$ the time where the first frame of the cecum is detected and $t_{groundtruth}$ the time corresponding to the first frame of the cecum according to the ground truth.

### 3.3 Cecum detection

The first experiment is the training of the cecum detection model. The model is trained and tested with only high-quality frames for the AO and the ICV since low-quality frames are mainly confusing the network. The negative frames are randomly taken from the whole insertion. The results for the detection task are shown in Table 2. The AO and ICV results are obtained by comparing the final predicted bounding box of the corresponding label (Fig. 4(f)) to the ground truth box (Fig. 4(b)). For the full procedures dataset, a specificity of 94% is reached for both AO and ICV while the sensitivity is only at 71%. The same observation can be made for the static frames with a specificity above 87% and a sensitivity below 77%. On the Kvasir dataset, however, the detection has better performance with a sensitivity above 93% and a specificity above 90%. Differences in performance between datasets can be explained by differences in image and view quality. The results combining both AO and ICV are obtained by checking if either AO or ICV are detected. The prediction is considered true if one of them is detected. This results in a better sensitivity (+ 4% on the static frames) but lower specificity (- 20% on the static frames) since both detections of AO and ICV are taken into account at the same time for each frame. Combining the AO and ICV detections helps to increase the sensitivity and is more similar to a real case usage where the objective is to detect the cecum, regardless of which specific structure is detected (AO or ICV). However, the specificity is impacted since a lot of false positives are added to the predictions. The per procedure sensitivity is computed only for the full procedures dataset since the two others are only static frames. A sensitivity of 92% is reached on the AO for only 78% on the ICV, meaning that 92% of the AOs and 78% of the ICVs are detected. However, when combining both of them, all the cecums are detected, either by detecting the AO or the ICV. The mean time to detection represents the mean number of frames between the first appearance of the AO or the ICV and its detection by the model. On average, one frame is needed for the AO while almost 14 frames are needed for the ICV. It is still a detection in less than 1 second so it does not have any impact on the withdrawal time. For the combined results, the mean time to detection is less than 4 frames.

**Table 2.** Results of AO and ICV detector. The first part concerns the static images datasets and the second part concerns the full procedures dataset and uses two more metrics only applicable for videos: the per procedure sensitivity and the mean time to detection.

	**Kvasir**		**Static frames**	
	Sensitivity	Specificity	Sensitivity	Specificity
	(per frame)	(per frame)	(per frame)	(per frame)
AO	93.84%	90.22%	76.76%	87.87%
ICV	N/A	98.83%	70.25%	94.44%
Both	93.85%	88.98%	80.21%	74.01%
**Full procedures**				
	Sensitivity	Specificity	Sensitivity	Mean time to detection
	(per frame)	(per frame)	(per procedure)	(in frames)

AO	74.44%	94.37%	92.86%	0.46
ICV	71.01%	96.66%	78.57%	13.82
Both	77.94%	91.11%	100%	3.38

### 3.4 Separation of the forcep model

The forcep class being a subclass of the therapeutic one, it can seem unnecessary to use a different classifier for it, but doing so improves the final model. The second experiment has been done to show the utility of using a separated forcep model on top of the therapeutic one. There are a few possibilities for the therapeutic classification:

• Use a therapeutic model trained with the forcep labels and use only 2 final models: cleaning and therapeutic.
• Use a therapeutic model trained without the forcep labels and use an independent forcep classifier.
• Use both a therapeutic model trained with the forcep labels and an independent forcep classifier.

**Fig. 7.**
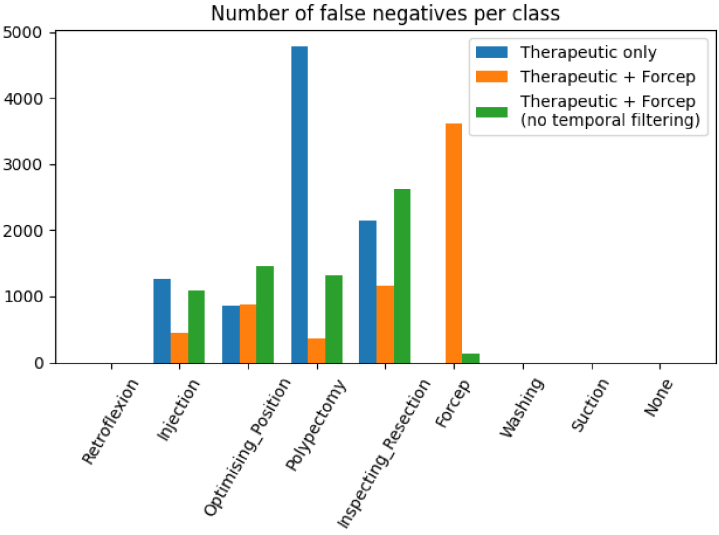
Distribution of false negatives for different therapeutic models for each subclass. Only the therapeutic subclasses have false negatives.

Figure 7 shows that the therapeutic model trained without the forcep labels has almost 5000 false negatives from the polypectomy subclass. This number drops to less than 500 when trained with the forcep frames. The number of false negatives from the other subclasses also decreases when trained with the forcep. This is the reason why the final therapeutic model includes the forcep subclass. But the last plot shows that without temporal filtering, there are fewer false negatives from the forcep class than with it. The temporal filtering successfully helps to reduce the number of false negatives for all the subclasses except the forcep one. This is due to the usage of the forcep during procedures. Forceps are used for two applications. One application is for biopsies to obtain a histological diagnosis. A separate application is for the therapeutic removal of polyps 3mm or less in size (polyps larger than this are removed with a snare). Both of these applications are non-exploratory phases, however, removal of polyps using biopsies can take a significantly longer time than random colonic biopsies, which typically take only a few seconds. Hence, it is important for the therapeutic classifier to detect forceps (for when polyps are removed <3mm in size) and for a separate classifier to detect biopsies which requires a much shorter temporal window. For all the other therapeutic subclasses, the labels are large continuous parts and the predictions can be smoothed very efficiently with a big temporal window. But for the forcep, a small temporal window is better than a large one. So to remove the false negatives added by the temporal filtering on the therapeutic class, an independent forcep model is used on top of the therapeutic one. That is why the forcep labels are used both in the therapeutic model and to train an independent forcep model.

### 3.5 Classification of forcep, therapeutic and cleaning phases

The final results for the classification task are shown in Table 3. The specificity is always better than the sensitivity for all the classes except for the therapeutic class where the sensitivity is 91.91% and the specificity is 84.97% before temporal filtering. A better specificity was the objective when adding more negative images to the training set. Since there are more negative frames than positive ones for every class, it is important to have a very high specificity to keep a low number of false positives. The lowest results are for the cleaning class with a sensitivity of 80.40% and a specificity of 88.76% before temporal filtering. A lot of images are similar to the negative class with either water or bubbles, which makes the cleaning class challenging. The suction subclass, in particular, is very hard to classify since there are a lot of edge cases that could be negative frames. The forcep classification achieves the highest specificity with 99.38% and sensitivity of 88.13%. Merging the models leads to an unavoidable decrease in specificity due to the logical OR used to combine the individual models. The false negatives from the therapeutic and cleaning models tend to be different and are then adding up when the models are combined. The sensitivity of the merged model is, in the worst-case scenario, similar to the lowest-performance model, cleaning in this case. But combining it with the therapeutic phase increases the sensitivity by more than 3%. The final model reaches a sensitivity of 85.87% and a specificity of 86.89%.

**Table 3.** Results of classification networks with and without temporal filtering.

	No temporal filtering		Temporal filtering	
	Sensitivity	Specificity	Sensitivity	Specificity
Therapeutic	91.91%	84.97%	94.25%	92.83%
Forcep	88.13%	99.38%	89.53%	99.70%
Cleaning	80.40%	88.76%	82.14%	92.05%
Merged model	N/A	N/A	85.87%	86.89%

### 3.6 Temporal filtering and discarding of uncertain predictions

**Fig. 8.**
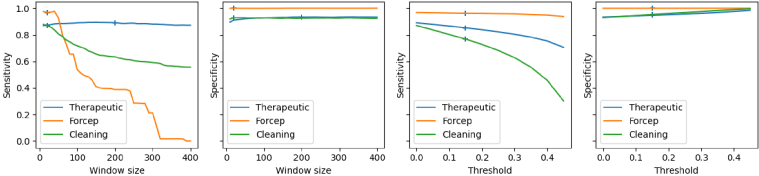
Evolution of sensitivity and specificity when changing the temporal window size and the threshold for the different models. Low sensitivity leads to an overestimation of the withdrawal time and a low specificity to an underestimation. The cross represents the chosen window size and the chosen threshold for each model: 20 and 0.15 for cleaning and forcep, and 200 and 0.15 for therapeutic.

This experiment aims to study the effect of the temporal window size on the results to use the most efficient one. To do this, the sensitivity and specificity are computed for different window sizes on the validation set. The final results with temporal filtering are computed using the best window according to Fig. 8. This figure shows that temporal filtering has different effects on each task. For cleaning, the specificity increases by almost 1% for the small windows getting rid of short false positives and then stays chaotic for bigger windows. The sensitivity decreases as the size of the window grows. Using a relatively small window (20 frames) is the best choice here. This allows keeping a sensitivity above 80% while taking advantage of the early jump in specificity. 20 is a good choice for the forcep as well since the behaviour is similar to the cleaning model. Particularly, with windows longer than 50 frames, the sensitivity drops suddenly while the specificity is high without the need for temporal filtering. Both cleaning and forcep are phases that last a few seconds and are alternating with other phases and negative frames. This is why a small temporal window maximises the performances of these two classes. Finally, for the therapeutic phase, both sensitivity and specificity are increased with large window sizes. Using a very large window is then the best choice for this model so 200 frames is used in the final results. The therapeutic phase usually lasts a few minutes, which explains the need for a big temporal window.

**Fig. 9.**
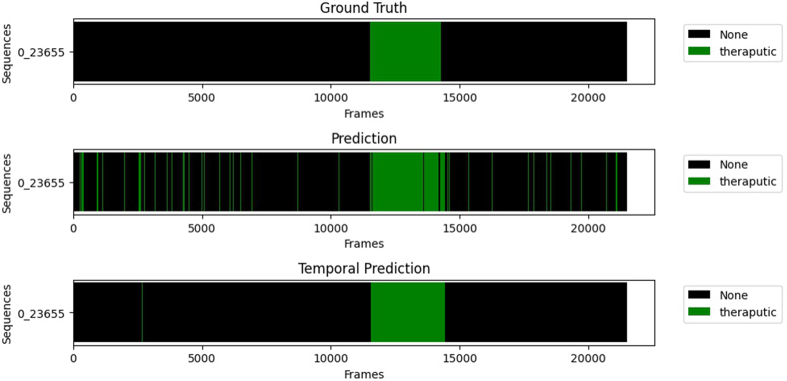
Example of the temporal filtering influence for a procedure on the therapeutic class. The green lines represent the positive frames (therapeutic) and the black ones the negative frames (any other class) for this model.

Temporal filtering improves both sensitivity and specificity for all the classes. The biggest improvement is the therapeutic specificity which goes from 84.97% to 92.83%. This is because the therapeutic phase is usually quite long and uninterrupted so it is safe to remove all the artefacts in the middle, which are mostly negative predictions. It also removes isolated therapeutic predictions which are very unlikely to be accurate. The gain in specificity is between 4% and less than 1% for the other classes. The sensitivity is also improved with a gain between 1% and 3% for all the classes. Figure 9 shows that the temporal filtering effectively helps to remove the small misclassifications, especially in the biggest classes therapeutic and cleaning or in the negative frames. But a lot of isolated mistakes cannot be corrected using this method. It also adds some errors when the frames of two classes are alternating. This is particularly the case with the cleaning class and the negative frames. This also explains the lower results for the cleaning sensitivity. A lot of cleaning frames and negative frames are very similar, which makes it harder for the network to differentiate them.

Figure 8 shows that, as for the temporal window size, the threshold has a big impact on the models depending on the class. Using a bigger rejection threshold increases a lot the specificity (from 93% to 99% for cleaning) at the cost of sensitivity (from 90% to 30% for cleaning). The biggest impact is on the cleaning class, followed by the therapeutic class. However, the impact of the threshold on the forcep is less than 1% for both sensitivity and specificity. This shows that the networks are very confident when classifying these classes whereas it is significantly harder for the cleaning and therapeutic classifiers. A threshold of 0.15 is used in the final estimation to keep a good balance between sensitivity and specificity.

### 3.7 Time estimation

**Fig. 10.**
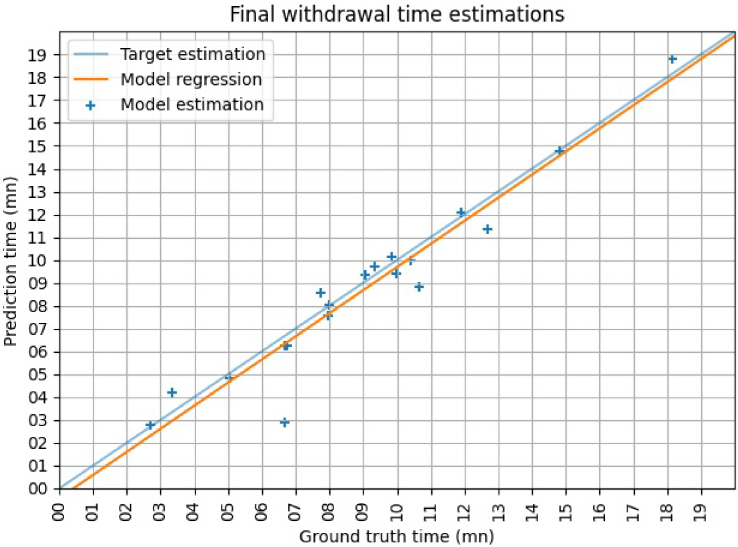
Final time estimations with target and predicted time. The x-axis represents the ground truth and the y-axis is the predictions. The perfect prediction would be equal to the ground truth, which is represented by the blue line. The closer to the target line the estimation is, the closer to the ground truth it is. The orange line represents the linear regression of the predictions.

The final withdrawal time estimation is computed by merging the results of the three classifiers. Each frame is classified as negative (included in withdrawal time) if and only if all three classifiers classify it as negative. If 0 is the negative class and 1 is positive, this is equivalent to a logical OR applied to the results, as shown in Fig. 5. The final classification is then a binary classification where the negative class will form the effective withdrawal. The time is computed based on a frame rate of 30 fps from the original procedures. The final time estimations are shown in Fig. 10. Each cross represents a different procedure. The closer to the target line the estimation is, the closer to the ground truth it is. The orange line is the linear regression of the predictions.

Out of 19 procedures, 18 are correctly classified as shorter or longer than 6 minutes. The time estimation for the fifth procedure, which is the one where the classification has a higher estimation error, is particularly far from the true value with a ground truth of 6:41 minutes and a prediction of 2:52 minutes. This is due to the presence of a lot of bubbles within the colon for most of the procedure. This infrequently occurs in clinical practice and is likely a result of a lack of training frames with bubbles present. For the second-worst results with a ground truth of 3:21 minutes and a prediction of 4:13 minutes, most of the errors are coming from the therapeutic and the cleaning class, which are the two hardest classes to classify and also the longest. If the recommendation of 6 minutes per withdrawal would need to be checked, 4 procedures would not meet the requirement according to the predictions. In this case, it is true for 3 procedures, the fourth one being the outlier one. For comparison purposes, the mean cleaning time per procedure is 2:59 minutes with a maximum of 5:01 minutes and a minimum of 1:01 minutes and the mean therapeutic time per procedure is 3:21 minutes with a maximum of 15:41 minutes and a minimum of 0:00 minutes. The final mean error for the 19 procedures is 5.52 seconds per minute for a mean time of 7:35 minutes per procedure.

## 4. Conclusion

This paper successfully proposes an automatic method to measure an accurate withdrawal time during a procedure. This method achieves a withdrawal time estimation with 18 out of 19 procedures correctly classified as shorter or longer than 6 minutes and a mean error of 5.52 seconds per minute. The AO/ICV detection results show good performance on static high-quality frames but can still be improved on the other static frames and the full procedures dataset. However, it is already possible to obtain a precise estimation of the withdrawal time and to find procedures where the 6 minutes target is not reached. Future work includes using more labelled data to improve the generalization of the networks and improving the AO/ICV detector. It would also be possible to explore some temporal networks for both classification and detection. Using more advanced architecture like a combination of CNN and RNN or HMM is also a promising possibility. Finally, it would be interesting to include motion estimation for both the detection and the phase classification to simplify the separation between insertion and withdrawal and to improve the precision of the withdrawal time estimation.

## Data Availability

Data underlying the results presented in this paper are not publicly available at this time but can be made available by a reasonable request that meets institutional ethical requirements. Data from the external dataset are available in Ref. [33].
